# Effects of the duration of bridge to lung transplantation with extracorporeal membrane oxygenation

**DOI:** 10.1371/journal.pone.0253520

**Published:** 2021-07-01

**Authors:** Dong Kyu Oh, Sang-Bum Hong, Tae Sun Shim, Dong Kwan Kim, Sehoon Choi, Geun Dong Lee, Won Kim, Seung-Il Park

**Affiliations:** 1 Department of Pulmonary and Critical Care Medicine, Asan Medical Center, University of Ulsan College of Medicine, Seoul, Republic of Korea; 2 Department of Cardiothoracic Surgery, Asan Medical Center, University of Ulsan College of Medicine, Seoul, Republic of Korea; 3 Department of Rehabilitation Medicine, Asan Medical Center, University of Ulsan College of Medicine, Seoul, Republic of Korea; Erasmus Medical Centre: Erasmus MC, NETHERLANDS

## Abstract

**Background:**

Although bridge to lung transplantation (BTT) with extracorporeal membrane oxygenation (ECMO) is increasingly performed, the impact of BTT and its duration on post-transplant outcomes are unclear.

**Methods:**

We retrospectively reviewed medical records of adult patients who underwent lung or heart-lung transplantation in our institution between January 2008 and December 2018. Data were compared in patients who did (n = 41; BTT) and did not (n = 36; non-BTT) require pre-transplant ECMO support. Data were also compared in patients who underwent short-term (<14 days; n = 21; ST-BTT) and long-term (≥14 days; n = 20; LT-BTT) BTTs.

**Results:**

Among 77 patients included, 51 (66.2%) were male and median age was 53 years. The median bridging time in the BTT group was 13 days (interquartile range [IQR], 7–19 days). Although simplified acute physiologic score II was significantly higher in the BTT group (median, 35; IQR, 31–49 in BTT group vs. median, 12; IQR, 7–19 in non-BTT group; *p*<0.001), 1-year (73.2% vs. 80.6%; p = 0.361) and 5-year (61.5% vs. 61.5%; p = 0.765) post-transplant survival rates were comparable in both groups. Comparison of ST- and LT-BTT subgroups showed that 1-year (90.5% vs. 55.0%; p = 0.009) and 5-year (73.0% vs. 48.1%; p = 0.030) post-transplant survival rates were significantly higher in ST-BTT group. In age and sex adjusted model, the LT-BTT was an independent risk factor for 1-year post-transplant mortality (hazard ratio, 3.019; 95% confidence interval, 1.119–8.146; p = 0.029), whereas the ST-BTT was not.

**Conclusions:**

Despite the severe illness, the BTT group showed favorable post-transplantation outcomes, particularly those bridged for less than 14 days.

## Introduction

Lung transplantation is a standard of care for patients with various non-malignant end-stage lung diseases [1]. However, the shortage of available donor lungs continues to be a huge problem. Waiting list mortality rates are as high as 121.8 deaths per 100 waitlist-years, with mortality rates being particularly high in patients with high severity indices [2]. These findings suggest that a conventional bridging strategy, consisting of invasive mechanical ventilation alone, may be suboptimal in severely ill patients. Rather, these patients may require additional cardiopulmonary support, such as extracorporeal membrane oxygenation (ECMO), while awaiting lung transplantation [3–5].

Traditionally, the benefit of ECMO as a bridge to lung transplantation (BTT) has been unclear because of the unfavorable clinical outcomes and the high complication rates [6, 7]. However, recent advances in ECMO systems and management strategies such as awaken and mobilized ECMO have improved the clinical outcomes of BTT over the last decade [2, 5, 6, 8, 9]. Meanwhile, the shortage of available donor lungs has prolonged bridging time to several weeks or even months [10–12]. Nonetheless, the effects of BTT and its duration on clinical outcomes in lung transplant recipients remain undetermined. The present study therefore retrospectively evaluated the impact of BTT and its duration on the post-transplant outcomes in patients who underwent lung transplantation.

## Materials and methods

### Study design and population

The study was performed at Asan Medical Center, a tertiary referral teaching hospital in Seoul, Republic of Korea. The data were retrospectively retrieved from adult patients aged ≥ 19 years who underwent lung or heart-lung transplantation at Asan Medical Center between January 2008 and December 2018. All data were automatically anonymized using our clinical data warehouse system [13]. The investigators had accessed fully anonymized data between March 2020 and March 2021. Patients who underwent multiorgan transplantation that did not include both the heart and lungs (e.g., liver-lung transplantation) were excluded. Included patients were followed until death or 31 December 2019. Patients were divided into those who did (BTT group) and did not (non-BTT group) require pre-transplant ECMO support. Moreover, patients in the BTT group were further subdivided into those undergoing short-term (< 14 days; ST-BTT group) and long-term (≥14 days; LT-BTT group) BTTs.

All organs used for transplantation in this study were provided by the government agency, the Korean Network for Organ Sharing (KONOS). The entire process for transplantation was strictly regulated by the relevant legislation. None of the transplant donors was from a vulnerable population and all donors or next of kin provided written informed consent that was freely given. The study protocol was approved by the institutional review board of Asan Medical Center (approval number 2020–0209) and the requirement for informed consent was waived because of the retrospective nature of the study and the use of anonymized clinical data.

### Lung transplantation protocol

Possible candidates for lung transplantation were selected according to the recommendations of the International Society for Heart and Lung Transplantation [6]. Patients with non-malignant end-stage lung diseases were considered for lung transplantation. The medical records of each candidate were reviewed by an institutional multidisciplinary lung transplantation committee consisting of pulmonologists, intensivists, cardiothoracic surgeons, infectious disease specialists, anesthesiologists, and radiologists, which confirmed the suitability of candidate for lung transplantation. Finally, the patient’s information was conveyed to the KONOS, and the candidate was listed for donor lung allocation according to the urgency status, which gives the most urgency priority (status 0) only to the patients requiring mechanical ventilation or ECMO [14].

Pre-transplantation management, including pulmonary rehabilitation, was performed primarily by pulmonologists. Upon transfer to the intensive care unit (ICU), the patient was managed by intensivists. Patients were regularly re-evaluated by the lung transplantation committee at least once per month. De-listing was considered if the patient had newly developed contraindications to lung transplantation, such as untreatable major organ dysfunction, uncorrectable bleeding diathesis, or limited functional status with poor rehabilitation potential [6].

When the donor lungs became available from the KONOS, the lung transplantation committee evaluated the condition of both donor and recipient and decided whether to perform the lung transplantation. Cardiopulmonary support during transplant surgery consisted of central veno-arterial (V-A) ECMO or cardiopulmonary bypass, as appropriate, with most patients weaned from the support at the end of transplantation. Patients who required continuing extracorporeal cardiopulmonary support were switched to V-A or veno-venous (V-V) ECMO, depending on the recipient’s condition, with support continued until the patient recovered or died.

### ECMO protocol

ECMO as a BTT was managed as recommended by the Extracorporeal Life Support Organization [15]. The indication for BTT was refractory hypoxemia, hypercarbia, or right heart failure despite optimal medical treatment in candidates for lung transplantation. Importantly, for patients with mechanical ventilation, the BTT was considered to facilitate awakening and mobilization, defined as at least standing with or without marching in place at bed side. Tracheostomy or extubation was performed within a few days from the ECMO support. However, when the donor lungs are available before tracheostomy or extubation, the lung transplantation was performed with endotracheal tube. Patients with BTT were mobilized as soon as possible to preserve the skeletal muscle mass. On the contrary, the BTT was not applied to patients 1) who did not require mechanical ventilation and 2) who could participate in rehabilitation with mechanical ventilation alone. On the other hand, the absolute contraindication for BTT was the patient not eligible for lung transplantation according to the standard criteria such as irreversible extrapulmonary end-organ damage or terminal illness. Those who were not expected to undergo adequate physical rehabilitation after the initiation of ECMO, as best predicted by the pre-ECMO performance status, were also contraindicated for BTT. Additional considerations included age > 65 years, limitations in vascular access, uncontrolled sepsis, coagulopathy, and prolonged mechanical ventilation. These patients could not survive until lung transplantation and were excluded from the present study.

Cannulation strategy was based on the described algorithm [3] along with additional considerations, including underlying diseases, respiratory and hemodynamic status, and anticipated worsening of hypoxemia or progressive secondary pulmonary hypertension with right ventricular dysfunction. Briefly, V-V ECMO was primarily considered for patients with hypoxemic respiratory failure without hemodynamic instability, whereas V-A ECMO was primarily considered for patients requiring hemodynamic support. Patients who developed refractory hemodynamic instability during V-V ECMO, due primarily to right heart failure, were switched to V-A or veno-arteriovenous (V-AV) ECMO, as appropriate. Initial cannulations were performed peripherally at the bedside in the ICU; if available, peripheral V-A or V-AV ECMO was switched to central V-A ECMO or right ventricular assist device with an oxygenator, called Oxy-RVAD, to facilitate physical rehabilitation [16, 17].

Two different ECMO systems were utilized, the QUADROX PLS System (Maquet Cardiopulmonary AG, Rastatt, Germany) and the CAPIOX EBS System (Terumo Cardiovascular Systems Corporation, Tokyo, Japan), with each system having its own oxygenator, pump, and console. Most patients were intravenously anticoagulated with unfractionated heparin, whereas those with confirmed or suspected heparin-induced thrombocytopenia were anticoagulated with argatroban until lung transplantation [18]. The dose of anticoagulant was titrated to yield a target activated partial thromboplastin time of 40–60 seconds. Attempts were made to awaken all patients and encourage them to participate in the rehabilitation program during ECMO support.

### Statistical analysis

Continuous variables were presented as median with interquartile range (IQR) and compared using Wilcoxon rank-sum tests. Categorical variables were presented as number (%) and compared using chi-square or Fisher’s exact test, as appropriate. Survival was calculated using the Kaplan-Meier method and compared using the log-rank test. A Cox proportional hazard regression model was used to assess the relationship between the independent variable and the post-transplantation mortality, with the hazard ratio (HR) used to quantify the association. Univariate analysis was initially performed to identify potentially significant risk factors with *p* < 0.10 for the multivariate analysis. The multicollinearity effects of risk factors were assessed using variance inflation factors with a cut-off level of > 10. All statistical analyses were two-sided, and *p* < 0.05 was considered significant. Statistical analysis was performed using the Statistical Package for Social Science (SPSS) version 22.0 for Windows (IBM Corporation, Armonk, NY, USA).

## Results

### Patient characteristics

During the study period, 181 patients were listed for lung or heart-lung transplantation (S1 Fig). Seventy-seven (42.5%) received transplantation, whereas 85 (47.0%) died on waiting list. The remaining 19 (10.5%) recovered without transplantation. The pre-transplant ECMO was initiated in 78 (43.1%) patients. Among them, 41 (52.6%) were successfully bridged to transplantation, 5 (6.4%) recovered without transplantation, and 32 (41.0%) died on waiting list. On the other hand, among patients who did not treated with pre-transplant ECMO (n = 103), 36 (35.0%) received transplantation, 14 (13.6%) recovered without transplantation, and 53 (51.4%) died on waiting list. The most common cause of death on waiting list was infection (n = 48; 56.5%) followed by progression of underlying disease (n = 32; 37.6%).

Among 77 patients who received transplantation, the median age was 53 years (IQR, 41–61 years) (Table 1) and male patients were 51 (66.2%). Twenty-seven (35.0%) patients died after transplantation, and the most common cause of death was infection (n = 12; 44.4%) followed by postoperative complication (n = 8; 29.6%) and chronic lung allograft dysfunction (n = 6; 22.2%). Ten (13.0%) patients underwent heart-lung transplantation and the remaining 66 (87.0%) underwent double-lung transplantation. All patients underwent initial transplantation, with none undergoing re-transplantation. The most common indication for lung transplantation was ILD in 37 (67.5%) patients, followed by bronchiolitis obliterans syndrome (BOS) in 7 (9.1%) patients, secondary to the previously treated hematologic malignancies. The median simplified acute physiologic score II (SAPS II) at the time of transplantation, was median 31 (IQR 12–39). Although 50 (64.9%) patients required invasive mechanical ventilation at the time of transplantation, 60 (77.9%) were mobilized with or without ECMO support (Table 1).

**Table 1:** Demographic and clinical characteristics of the BTT and the non-BTT groups at the time of transplantation.

	All (n = 77)	BTT (n = 41)	Non-BTT (n = 36)	P-value
Age, years, median (IQR)	53 (41–61)	55 (41–62)	51 (40–60)	0.358
Male, n (%)	51 (66.2)	26 (63.4)	25 (69.4)	0.577
BMI, kg/m^2^, median (IQR)	22.6 (19.4–24.9)	23.1 (20.2–25.4)	21.6 (16.7–24.4)	0.109
Blood type, n (%)				0.655
A	27 (35.1)	17 (41.5)	10 (27.8)	
B	23 (29.9)	11 (26.8)	12 (33.3)	
O	14 (18.2)	7 (17.1)	7 (19.4)	
AB	13 (16.9)	6 (14.6)	7 (19.4)	
Diagnosis, n (%)				0.479
Interstitial lung disease	52 (67.5)	30 (73.2)	22 (61.1)	
Bronchiolitis obliterans syndrome^a^	7 (9.1)	2 (4.9)	5 (13.9)	
Pulmonary vascular disease	5 (6.5)	3 (7.3)	2 (5.6)	
Others	13 (16.9)	6 (14.6)	7 (19.4)	
SAPS II, median (IQR)	31 (12–39)	35 (31–49)	12 (7–19)	<0.001
Type of transplantation				0.742
Double-lung	67 (87.0)	35 (85.4)	32 (88.9)	
Heart-lung	10 (13.0)	6 (14.6)	4 (11.1)	
Mechanical ventilation, n (%)				<0.001
Not required	27 (35.1)	1 (2.4)	26 (72.2)	
Intubated	13 (16.9)	10 (24.4)	3 (8.3)	
Tracheostomized	37 (48.1)	30 (73.2)	7 (19.4)	
Mobilization, n (%)	60 (77.9)	31 (75.6)	29 (80.6)	0.602
Time to lung transplantation, days, median (IQR)				
From hospital admission	16 (1–39)	29 (14–42)	1 (0–25)	<0.001
From ICU admission	10 (0–25)	16 (10–30)	0 (0–2)	<0.001
Ischemic time, h, median (IQR)	4.1 (3.8–4.5)	4.1 (3.9–4.6)	4.0 (3.7–4.5)	0.231
Primary graft dysfunction at 72 hours, n (%)				0.124
Grade 0 or 1	26 (33.8)	11 (26.8)	15 (41.7)	
Grade 2	38 (49.4)	20 (48.8)	18 (50.0)	
Grade 3	13 (16.9)	10 (24.4)	3 (8.3)	
AKI requiring dialysis, n (%)	18 (23.4)	11 (26.8)	7 (19.4)	0.445
Hypotension requiring vasopressors for > 24 hours, n (%)	17 (22.1)	12 (29.3)	5 (13.9)	0.105

AKI, acute kidney injury; BMI, body mass index; BTT, bridge to lung transplantation; IQR, interquartile range; SAPS II, simplified acute physiologic score II.

^a^Secondary to the previously treated hematologic malignancies.

### Comparison of variables between the BTT and the non-BTT groups

Of the 77 patients included, 41 (53.2%) underwent BTT with ECMO (BTT group), whereas 36 (46.8%) did not (non-BTT group) (Table 1). The median SAPS II (35; IQR, 31–49 vs. 12; IQR, 7–19; p < 0.001) and the percentage of patients requiring invasive mechanical ventilation (97.6% vs. 27.8%; p < 0.001) at the time of transplantation were significantly higher in the BTT group. Furthermore, the median pre-transplant lengths of stay in the hospital (29 days; IQR, 14–42 days vs. 1 day; IQR, 0–25 days; p < 0.001) and in the ICU (10 days; IQR, 0–25 days vs. 0 days; IQR, 0–2 days; p < 0.001) were significantly longer in the BTT group. However, the proportion of mobilized patients was comparable in both groups (75.6% vs. 80.6%; p = 0.602).

**Fig 1:**
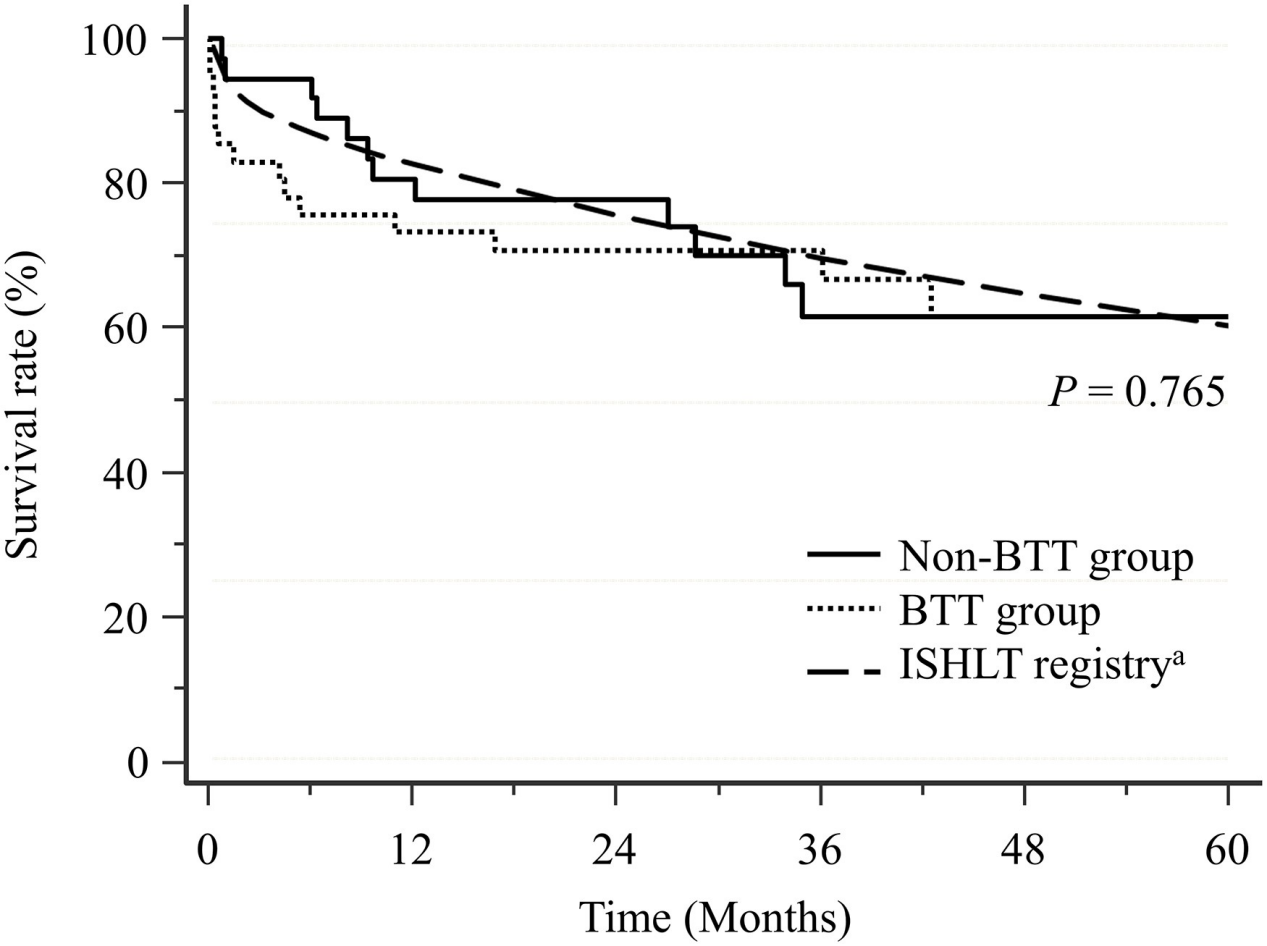
Kaplan-Meier analysis of post-transplant survival in the BTT and the non-BTT groups. BTT, bridge to lung transplantation; ISHLT, International society for heart and lung transplantation. ^a^Data from the International Thoracic Organ Transplant Registry of the International Society for Heart and Lung Transplantation [19].

The overall 1-year, 3-year, and 5-year post-transplant survival rates were 76.6%, 66.3%, and 61.9%, respectively and were comparable to those of international adult lung transplantation registry (83.2%, 70.2%, and 60.7%, respectively) (Fig 1). The 1-year (73.2% vs. 80.6%; p = 0.361), 3-year (70.6% vs. 61.5%; p = 0.936), and 5-year (61.5% vs. 61.5%; p = 0.765) post-transplant survival rates were comparable in both groups and were similar to those from the International Society for Heart and Lung Transplantation registry (83.2%, 70.2%, and 60.7%, respectively) (Fig 1) [19].

### Comparison of variables between the short- and the long-term BTT groups

**Fig 2:**
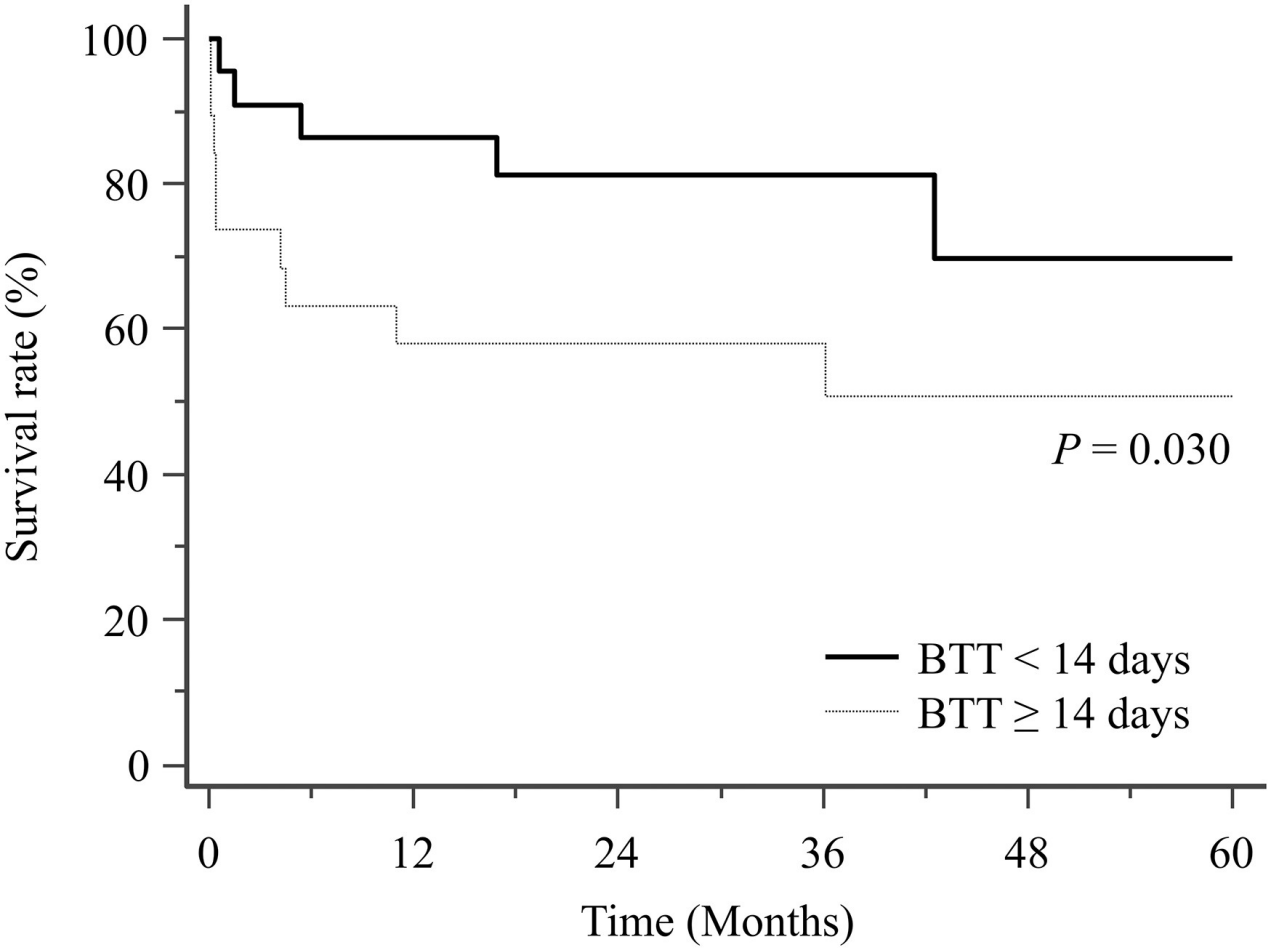
Kaplan-Meier analysis of post-transplantation survival in the short-term (< 14 days) and the long-term (≥ 14 days) BTT groups. BTT, bridge to lung transplantation.

The 41 patients successfully bridged to lung transplantation had a median bridging time of 13 days (IQR, 7–19). Of these 41 patients, 21 (51.2%) required pre-transplant ECMO support for < 14 days (ST-BTT group) and 20 (48.8%) required for ≥ 14 days (LT-BTT group) (Table 2). Despite their comparable SAPS II (median, 34; IQR, 33–40 in ST-BTT group vs. median, 36; IQR, 29–56 in LT-BTT group; p = 0.666) and proportion of mobilized patients (81.0% vs. 70.0%; p = 0.484), hemodynamic support with V-A ECMO was less frequently required in the ST-BTT group (14.3% vs. 45.0%; p = 0.031). Moreover, 1-year (90.5% vs. 55.0%; p = 0.009), 3-year (85.2% vs. 55.0%; p = 0.022), and 5-year (73.0% vs. 48.1%; p = 0.030) post-transplant survival rates were significantly higher in the ST-BTT group (Fig 2). In addition, the rate of ECMO-related hemorrhagic complications was significantly lower in the ST-BTT group (14.3% vs. 50.0%; p = 0.014) and the ST-BTT group required fewer blood transfusions (Table 2).

**Table 2:** Demographic and clinical characteristics of the short-term (< 14 days) and the long-term (≥ 14 days) BTT groups at the time of transplantation.

	Short-term (n = 21)	Long-term (n = 20)	P-value
Age, years, median (IQR)	60 (48–63)	51 (39–57)	0.053
Male, n (%)	13 (61.9)	13 (65.0)	0.837
BMI, kg/m^2^, median (IQR)	22.6 (20.7–24.8)	23.1 (19.9–26.1)	0.876
Blood type, n (%)			0.333
A	7 (33.3)	10 (50.0)	
B	5 (23.8)	6 (30.0)	
O	4 (19.0)	3 (15.0)	
AB	5 (23.8)	1 (5.0)	
Diagnosis, n (%)			0.286
Interstitial lung disease	16 (76.2)	14 (70.0)	
Bronchiolitis obliterans syndrome^a^	1 (4.8)	1 (5.0)	
Pulmonary vascular disease	0 (0.0)	3 (15.0)	
Others	4 (19.0)	2 (10.0)	
SAPS II, median (IQR)	34 (33–40)	36 (29–56)	0.666
Type of transplantation, n (%)			0.410
Double lung	19 (90.5)	16 (80.0)	
Heart-lung	2 (9.5)	4 (20.0)	
Mechanical ventilation, n (%)			0.056
Not required	1 (4.8)	0 (0.0)	
Intubated	8 (38.1)	2 (10.0)	
Tracheostomized	12 (57.1)	18 (90.0)	
Mobilization, n (%)	17 (81.0)	14 (70.0)	0.484
Initial configuration, n (%)			0.093
Veno-venous	20 (95.2)	15 (75.0)	
Veno-arterial	1 (4.8)	5 (25.0)	
Configuration at transplantation, n (%)			0.031
Veno-venous	18 (85.7)	11 (55.0)	
Veno-arterial	3 (14.3)	9 (45.0)	
Configuration change, n (%)	5 (23.8)	10 (50.0)	0.082
Time to ECMO initiation, days, median (IQR)			
From hospital admission	10 (4–25)	15 (5–27)	0.513
From ICU admission	2 (0–10)	3 (0–11)	0.821
Time to lung transplantation, days, median (IQR)			
From hospital admission	15 (10–32)	40 (28–50)	0.001
From ICU admission	11 (8–16)	29 (18–39)	<0.001
From ECMO initiation	8 (3–11)	19 (14–31)	<0.001
Complications during bridging, n (%)			
Bleeding complications	3 (14.3)	10 (50.0)	0.014
Thrombotic complications	3 (14.3)	6 (30.0)	0.277
Renal replacement therapy	2 (9.5)	3 (15.0)	0.663
Transfusion, units, median (IQR)			
Red blood cell	3 (2–12)	15 (9–22)	0.001
Platelet	0 (0–2)	0 (0–9)	0.134
Fresh frozen plasma	0 (0–8)	21 (0–59)	0.013
Cryoprecipitate	0 (0–0)	0 (0–5)	0.235
Ischemic time, h, median (IQR)	4.1 (4.0–4.4)	4.3 (3.8–4.9)	0.359
Primary graft dysfunction at 72 hours, n (%)			0.716
Grade 0 or 1	6 (28.6)	6 (25.0)	
Grade 2	11 (52.4)	9 (45.0)	
Grade 3	4 (19.0)	6 (30.0)	
AKI requiring dialysis, n (%)	4 (19.0)	7 (35.0)	0.249
Hypotension requiring vasopressors for > 24 hours, n (%)	5 (23.8)	7 (35.0)	0.431

AKI, acute kidney injury; BMI, body mass index; BTT, bridge to lung transplantation; ECMO, extracorporeal membrane oxygenation; IQR, interquartile range; SAPS II, simplified acute physiologic score II.

^a^Secondary to the previously treated hematologic malignancies.

### Risk factors for post-transplant mortality

Univariate analysis showed that BTT group was not associated with 1-, 3-, and 5-year post-transplant mortalities (S1–S3 Tables). However, a comparison of the BTT subgroups (LT-BTT and ST-BTT) with the non-BTT group showed that the LT-BTT was a significant risk factor for 1-year post-transplant mortality (hazard ratio [HR], 3.070; 95% confidence interval [CI], 1.141–8.260; p = 0.026), whereas the ST-BTT was not (HR, 0.481; 95% CI, 0.100–2.315; p = 0.361) (S1 Table). Adjusted for age and sex, the LT-BTT remained significantly associated with 1-year post-transplant mortality (HR, 3.019; 95% CI, 1.119–8.146; p = 0.029), whereas the ST-BTT was not (HR, 0.464; 95% CI, 0.094–2.291; p = 0.346) (Table 3). Neither LT-BTT nor ST-BTT was significantly associated with 3- and 5-year post-transplant survivals when compared with the non-BTT group (S2 and S3 Tables).

**Table 3:** Risk factors for 1-year post-transplant mortality.

Variables	Adjusted HR^a^	95% CI	P-value
Age	1.004	0.961–1.050	0.846
Male	0.795	0.257–2.464	0.691
BTT			0.017
Non-BTT	Reference	Reference	Reference
Short-term BTT	0.464	0.094–2.291	0.346
Long-term BTT	3.019	1.119–8.146	0.029

BTT, bridge to lung transplantation; CI, confidence interval; HR = hazard ratio.

^a^Adjusted for age, sex, and BTT.

## Discussion

This retrospective study investigated the impact of BTT and its duration on the post-transplant outcomes. Despite having more severe illness, the BTT group showed post-transplant outcomes comparable with those of the non-BTT group, with BTT itself not being a risk factor for post-transplant mortality. However, when the bridged patients were further divided based on the duration of bridging, the LT-BTT group, bridged for ≥ 14 days, showed a worse prognosis than the ST-BTT group, bridged for < 14 days, with LT-BTT being an independent risk factor for 1-year post-transplant mortality.

ECMO may be used as a bridging therapy for lung transplantation in patients with severe respiratory failure. Although the initial experience was discouraging, with high rates of death and complications [6, 7], studies in the last decade have reported promising results, with 1-year post-transplant survival rate ranging from 54% to 93% [3, 4, 9, 20–25]. Our results, showing that patients in the BTT group had 1-, 3-, and 5-year post-transplant survival rates of 73.2%, 70.6%, and 61.5%, respectively, are consistent with the previous results, confirming that the ECMO may be used as a BTT in selected patients.

At present, however, characteristics of patients who may benefit from this potentially life-saving procedure remain unclear. Although several articles have suggested factors that may be associated with favorable post-transplant outcomes, such as young age (e.g., < 35 to 50 years), low severity index (e.g., SOFA score < 6), and a good potential for physical activity [3, 4, 6, 21–27], these are not absolute inclusion or exclusion criteria for BTT. Furthermore, rapid developments in this field may result in easing of the strict criteria for BTT. For example, in this study, the median age of patients in the BTT group was 55 years, with this group being much older than in the previous studies, in which median age ranged from 31–45 years [3, 4, 20–25]. In addition, the median baseline SAPS II in our BTT group, 35 (IQR, 31–49), was much higher than that reported previously, 24 (IQR, 19–32) [25]. The high proportion of mobilized patients in our BTT group, 75.6%, and their favorable post-transplant outcomes, suggest that older and more severely ill patients may also benefit from BTT, especially if they are physically active. Additional studies are required to confirm these findings and to expand the possible inclusion criteria for BTT.

It is also necessary to determine the association of bridging time with post-transplant outcomes. Because of the shortage of donor lungs, bridging time is often extended to several weeks or even months [10–12]. At present, however, the impact of bridging time on post-transplant outcomes and the specific time limit for ECMO support are undetermined [27]. In this study, the long-term bridging for ≥ 14 days resulted in 1-, 3-, and 5-year survival rates of 55.0%, 55.0%, and 48.1%, respectively, which were significantly worse than the short-term bridging for < 14 days, 90.5%, 85.2%, and 73.0%, respectively. Moreover, a bridging time ≥ 14 days was an independent risk factor for 1-year post-transplant mortality. These findings are consistent with those of a previous study, which reported the 1-year post-transplant survival rate of 50% in patients bridged for > 14 days and 100% in patients bridged for ≤ 14 days [20]. Furthermore, in a recent study, Langer and colleagues reported that the duration of BTT < 30 days is associated with better post-transplant outcomes and suggested the cut-off of 30 days [28]. Although the specific cut-off may vary depending on the expertise of each institution, it needs to be emphasized that the duration of BTT may impact on the post-transplant outcomes.

Nonetheless, caution should be exercised when interpreting these results because the factors that contribute to the bridging time associated differences in clinical outcomes remain unclear. Although worsening of illness during long-term BTT may be associated with worse post-transplant outcomes [20], our study found that SAPS II at the time of transplantation were comparable in the groups. Other risk factors such as infection, deconditioning, and ECMO complications also may contribute to the poor prognosis in the long-term BTT group [29]. In this study, interestingly, the requirement for V-A ECMO was more frequent in the LT-BTT group. Moreover, ECMO configuration changes tended to be more frequent in the LT-BTT group. Because switching from V-V to V-A ECMO in patients who developed refractory hemodynamic instability was regarded as mostly due to right heart failure, the worse prognosis of patients in the LT-BTT group may be associated with the worsening of pulmonary hypertension and/or the development of *cor pulmonale* during BTT. Although studies have suggested that severe pulmonary hypertension and *cor pulmonale* are associated with unfavorable outcomes in patients who undergo BTT [23, 27], this could not be verified in this retrospective study because all patients did not routinely undergo echocardiography and right heart catheterization. Additional studies are needed to elucidate this possibility.

This study had several limitations. First, it was a retrospective non-interventional study performed at a single center. Therefore, it may have inherent biases and the results may not be applicable to other lung transplantation centers, particularly if their expertise in the performance of ECMO and lung transplantation differs. Second, the regional differences in lung allocation system and the consequent high proportion of BTT group in this study may make our results difficult to apply to other centers. In Korea, the lungs are allocated according to the recipient’s severity of illness and those with mechanical ventilation and/or ECMO receive the highest priority for the lungs [14]. Therefore, in our system, the recipient’s severity of illness had increased consistently and, nowadays more than 60% of recipients require mechanical ventilation and/or ECMO in the pre-transplant period [30]. This circumstance contributed to the exceptionally high proportion of BTT in this study and may limit the generalization of our findings. Third, because of the extremely low incidence of cystic fibrosis in east Asian countries [31], they were not included in the present study. Because they are known to be most beneficial from BTT, the exclusion of patients with cystic fibrosis might bias the results. Fourth, no single lung transplantation was included in the study. Considering the differences in the post-transplant outcomes between single and double lung transplantations, the results need to be interpreted with caution. Fifth, although this study was designed to investigate post-transplant outcomes and relevant factors in patients who underwent BTT, only patients who underwent lung transplantation were included. Excluding patients who failed to bridge to lung transplantation may constitute a selection bias. Lastly, because the study period was over 10 years, a learning curve bias cannot be excluded.

## Conclusions

Despite the severe illness, in our institution, patients bridged to lung transplantation with ECMO showed favorable post-transplant outcomes, particularly those bridged for < 14 days. These suggest that the current strict criteria for BTT may be eased in selected patients, allowing more patients to benefit from this potentially life-saving procedure.

## Supporting information

S1 Fig Patient flow. BTT, bridge to lung transplantation. ^a^BTT group; ^b^Non-BTT group; ^c^Short-term BTT group; ^d^Long-term BTT group.
(TIF)

S1 Table Risk factors for 1-year post-transplant mortality in univariate analysis. BTT, bridge to lung transplantation; CI, confidence interval; ECMO, extracorporeal membrane oxygenation; HR, hazard ratio; SAPS II, simplified acute physiologic score II.
(DOCX)

S2 Table Risk factors for 3-year post-transplant mortality. BTT, bridge to lung transplantation; CI, confidence interval; ECMO, extracorporeal membrane oxygenation; HR, hazard ratio; SAPS II, simplified acute physiologic score II. ^a^Adjusted for age, sex, and BTT.
(DOCX)

S3 Table Risk factors for 5-year post-transplant mortality. BTT, bridge to lung transplantation; CI, confidence interval; ECMO, extracorporeal membrane oxygenation; HR, hazard ratio; SAPS II, simplified acute physiologic score II. ^a^Adjusted for age, sex, and BTT.
(DOCX)

## References

[1] Kotloff RM, Thabut G. Lung transplantation. American journal of respiratory and critical care medicine. 2011;184(2):159–71. Epub 2011/04/08. doi: 10.1164/rccm.201101-0134CI . 21471083

[2] Valapour M, Lehr CJ, Skeans MA, Smith JM, Uccellini K, Goff R, et al. OPTN/SRTR 2018 Annual Data Report: Lung. American journal of transplantation: official journal of the American Society of Transplantation and the American Society of Transplant Surgeons. 2020;20 Suppl s1:427–508. Epub 2020/01/04. doi: 10.1111/ajt.15677 . 31898416

[3] Hoetzenecker K, Donahoe L, Yeung JC, Azad S, Fan E, Ferguson ND, et al. Extracorporeal life support as a bridge to lung transplantation-experience of a high-volume transplant center. The Journal of thoracic and cardiovascular surgery. 2018;155(3):1316–28.e1. Epub 2017/12/19. doi: 10.1016/j.jtcvs.2017.09.161 . 29248282

[4] Biscotti M, Gannon WD, Agerstrand C, Abrams D, Sonett J, Brodie D, et al. Awake Extracorporeal Membrane Oxygenation as Bridge to Lung Transplantation: A 9-Year Experience. The Annals of thoracic surgery. 2017;104(2):412–9. Epub 2017/03/01. doi: 10.1016/j.athoracsur.2016.11.056 . 28242078

[5] Chiumello D, Coppola S, Froio S, Colombo A, Del Sorbo L. Extracorporeal life support as bridge to lung transplantation: a systematic review. Crit Care. 2015;19(1):19. Epub 2015/03/17. . 25774818 10.1186/s13054-014-0686-7 PMC4302424

[6] Weill D, Benden C, Corris PA, Dark JH, Davis RD, Keshavjee S, et al. A consensus document for the selection of lung transplant candidates: 2014--an update from the Pulmonary Transplantation Council of the International Society for Heart and Lung Transplantation. The Journal of heart and lung transplantation: the official publication of the International Society for Heart Transplantation. 2015;34(1):1–15. Epub 2014/08/03. doi: 10.1016/j.healun.2014.06.014 . 25085497

[7] Diaz-Guzman E, Hoopes CW, Zwischenberger JB. The evolution of extracorporeal life support as a bridge to lung transplantation. ASAIO journal (American Society for Artificial Internal Organs: 1992). 2013;59(1):3–10. Epub 2012/12/29. doi: 10.1097/MAT.0b013e31827461c2 . 23271390

[8] Brodie D, Slutsky AS, Combes A. Extracorporeal Life Support for Adults With Respiratory Failure and Related Indications: A Review. Jama. 2019;322(6):557–68. Epub 2019/08/14. doi: 10.1001/jama.2019.9302 . 31408142

[9] Fuehner T, Kuehn C, Hadem J, Wiesner O, Gottlieb J, Tudorache I, et al. Extracorporeal membrane oxygenation in awake patients as bridge to lung transplantation. Am J Respir Crit Care Med. 2012;185(7):763–8. Epub 2012/01/24. doi: 10.1164/rccm.201109-1599OC . 22268135

[10] Salam S, Kotloff R, Garcha P, Krishnan S, Joshi D, Grady P, et al. Lung Transplantation After 125 Days on ECMO for Severe Refractory Hypoxemia With No Prior Lung Disease. ASAIO journal (American Society for Artificial Internal Organs: 1992). 2017;63(5):e66–e8. Epub 2017/09/01. doi: 10.1097/MAT.0000000000000450 . 28857906

[11] Broome M, Palmer K, Schersten H, Frenckner B, Nilsson F. Prolonged extracorporeal membrane oxygenation and circulatory support as bridge to lung transplant. The Annals of thoracic surgery. 2008;86(4):1357–60. Epub 2008/09/23. doi: 10.1016/j.athoracsur.2008.03.053 . 18805197

[12] Kon ZN, Wehman PB, Gibber M, Rabin J, Evans CF, Rajagopal K, et al. Venovenous extracorporeal membrane oxygenation as a bridge to lung transplantation: successful transplantation after 155 days of support. The Annals of thoracic surgery. 2015;99(2):704–7. Epub 2015/02/03. doi: 10.1016/j.athoracsur.2014.04.097 . 25639416

[13] Shin SY, Park YR, Shin Y, Choi HJ, Park J, Lyu Y, et al. A De-identification method for bilingual clinical texts of various note types. Journal of Korean medical science. 2015;30(1):7–15. Epub 2015/01/02. doi: 10.3346/jkms.2015.30.1.7 . 25552878 PMC4278030

[14] Yu WS, Kim SY, Kim YT, Lee HJ, Park S, Choi SM, et al. Characteristics of Lung Allocation and Outcomes of Lung Transplant according to the Korean Urgency Status. Yonsei Med J. 2019;60(10):992–7. Epub 2019/09/21. doi: 10.3349/ymj.2019.60.10.992 . 31538435 PMC6753335

[15] Tonna JE, Abrams D, Brodie D, Greenwood JC, Rubio Mateo-Sidron JA, Usman A, et al. Management of Adult Patients Supported with Venovenous Extracorporeal Membrane Oxygenation (VV ECMO): Guideline from the Extracorporeal Life Support Organization (ELSO). Asaio j. 2021;67(6):601–10. Epub 2021/05/10. doi: 10.1097/MAT.0000000000001432 . 33965970 PMC8315725

[16] Oh DK, Shim TS, Jo KW, Park SI, Kim DK, Choi S, et al. Right ventricular assist device with an oxygenator using extracorporeal membrane oxygenation as a bridge to lung transplantation in a patient with severe respiratory failure and right heart decompensation. Acute Crit Care. 2019. Epub 2019/11/20. doi: 10.4266/acc.2018.00416 . 31743636 PMC7280790

[17] Nam KH, Koh Y, Lim CM, Huh JW, Jung SH, Kang PJ, et al. Central Extracorporeal Membrane Oxygenation for Bridging of Right-Sided Heart Failure to Lung Transplantation: A Single-Center Experience and Literature Review. J Cardiothorac Vasc Anesth. 2019;33(7):1873–6. Epub 2019/03/23. doi: 10.1053/j.jvca.2019.01.059 . 30898420

[18] Oh DK, Kim DK, Choi S, Hong SB. Preoperative and intraoperative argatroban as anticoagulant for bridging a patient with heparin-induced thrombocytopaenia to lung transplantation. European journal of cardio-thoracic surgery: official journal of the European Association for Cardio-thoracic Surgery. 2019. Epub 2019/10/28. doi: 10.1093/ejcts/ezz294 . 31651940

[19] Khush KK, Potena L, Cherikh WS, Chambers DC, Harhay MO, Hayes D Jr., et al. The International Thoracic Organ Transplant Registry of the International Society for Heart and Lung Transplantation: 37th adult heart transplantation report-2020; focus on deceased donor characteristics. J Heart Lung Transplant. 2020;39(10):1003–15. Epub 2020/08/20. doi: 10.1016/j.healun.2020.07.010 . 32811772 PMC7737223

[20] Crotti S, Iotti GA, Lissoni A, Belliato M, Zanierato M, Chierichetti M, et al. Organ allocation waiting time during extracorporeal bridge to lung transplant affects outcomes. Chest. 2013;144(3):1018–25. Epub 2013/04/20. doi: 10.1378/chest.12-1141 . 23599162

[21] Hoopes CW, Kukreja J, Golden J, Davenport DL, Diaz-Guzman E, Zwischenberger JB. Extracorporeal membrane oxygenation as a bridge to pulmonary transplantation. The Journal of thoracic and cardiovascular surgery. 2013;145(3):862–7; discussion 7–8. Epub 2013/01/15. doi: 10.1016/j.jtcvs.2012.12.022 . 23312979

[22] Benazzo A, Schwarz S, Frommlet F, Schweiger T, Jaksch P, Schellongowski P, et al. Twenty-year experience with extracorporeal life support as bridge to lung transplantation. The Journal of thoracic and cardiovascular surgery. 2019;157(6):2515–25.e10. Epub 2019/03/30. doi: 10.1016/j.jtcvs.2019.02.048 . 30922636

[23] Weig T, Irlbeck M, Frey L, Zwissler B, Winter H, Preissler G, et al. Parameters associated with short- and midterm survival in bridging to lung transplantation with extracorporeal membrane oxygenation. Clin Transplant. 2013;27(5):E563–70. Epub 2013/08/01. doi: 10.1111/ctr.12197 . 23898897

[24] Inci I, Klinzing S, Schneiter D, Schuepbach RA, Kestenholz P, Hillinger S, et al. Outcome of Extracorporeal Membrane Oxygenation as a Bridge To Lung Transplantation: An Institutional Experience and Literature Review. Transplantation. 2015;99(8):1667–71. Epub 2015/08/27. doi: 10.1097/TP.0000000000000653 . 26308302

[25] Tipograf Y, Salna M, Minko E, Grogan EL, Agerstrand C, Sonett J, et al. Outcomes of Extracorporeal Membrane Oxygenation as a Bridge to Lung Transplantation. Ann Thorac Surg. 2019;107(5):1456–63. Epub 2019/02/23. doi: 10.1016/j.athoracsur.2019.01.032 . 30790550

[26] Hayanga AJ, Aboagye J, Esper S, Shigemura N, Bermudez CA, D’Cunha J, et al. Extracorporeal membrane oxygenation as a bridge to lung transplantation in the United States: an evolving strategy in the management of rapidly advancing pulmonary disease. The Journal of thoracic and cardiovascular surgery. 2015;149(1):291–6. Epub 2014/12/20. doi: 10.1016/j.jtcvs.2014.08.072 . 25524684

[27] Loor G, Simpson L, Parulekar A. Bridging to lung transplantation with extracorporeal circulatory support: when or when not? Journal of thoracic disease. 2017;9(9):3352–61. Epub 2017/12/10. doi: 10.21037/jtd.2017.08.117 . 29221320 PMC5708444

[28] Langer F, Aliyev P, Schäfers HJ, Trudzinski FC, Seiler F, Bals R, et al. Improving Outcomes in Bridge-to-Transplant: Extended Extracorporeal Membrane Oxygenation Support to Obtain Optimal Donor Lungs for Marginal Recipients. Asaio j. 2019;65(5):516–21. Epub 2018/07/26. doi: 10.1097/MAT.0000000000000843 . 30044239

[29] Nasir BS, Klapper J, Hartwig M. Lung Transplant from ECMO: Current Results and Predictors of Post-transplant Mortality. Curr Transplant Rep. 2021:1–11. Epub 2021/04/13. doi: 10.1007/s40472-021-00323-4 . 33842193 PMC8021937

[30] Ko RE, Lee JG, Kim SY, Kim YT, Choi SM, Kim DH, et al. Extracorporeal membrane oxygenation as a bridge to lung transplantation: analysis of Korean organ transplantation registry (KOTRY) data. Respir Res. 2020;21(1):20. Epub 2020/01/15. doi: 10.1186/s12931-020-1289-2 . 31931798 PMC6958687

[31] Singh M, Rebordosa C, Bernholz J, Sharma N. Epidemiology and genetics of cystic fibrosis in Asia: In preparation for the next-generation treatments. Respirology. 2015;20(8):1172–81. Epub 2015/10/07. doi: 10.1111/resp.12656 . 26437683

