# Supplementary figures and images for: Effects of the duration of bridge to lung transplantation with extracorporeal membrane oxygenation
Source: PLoS One. 2021 Jul 1;16(7):e0253520. doi: 10.1371/journal.pone.0253520 (PMC8248733; doi:10.1371/journal.pone.0253520)

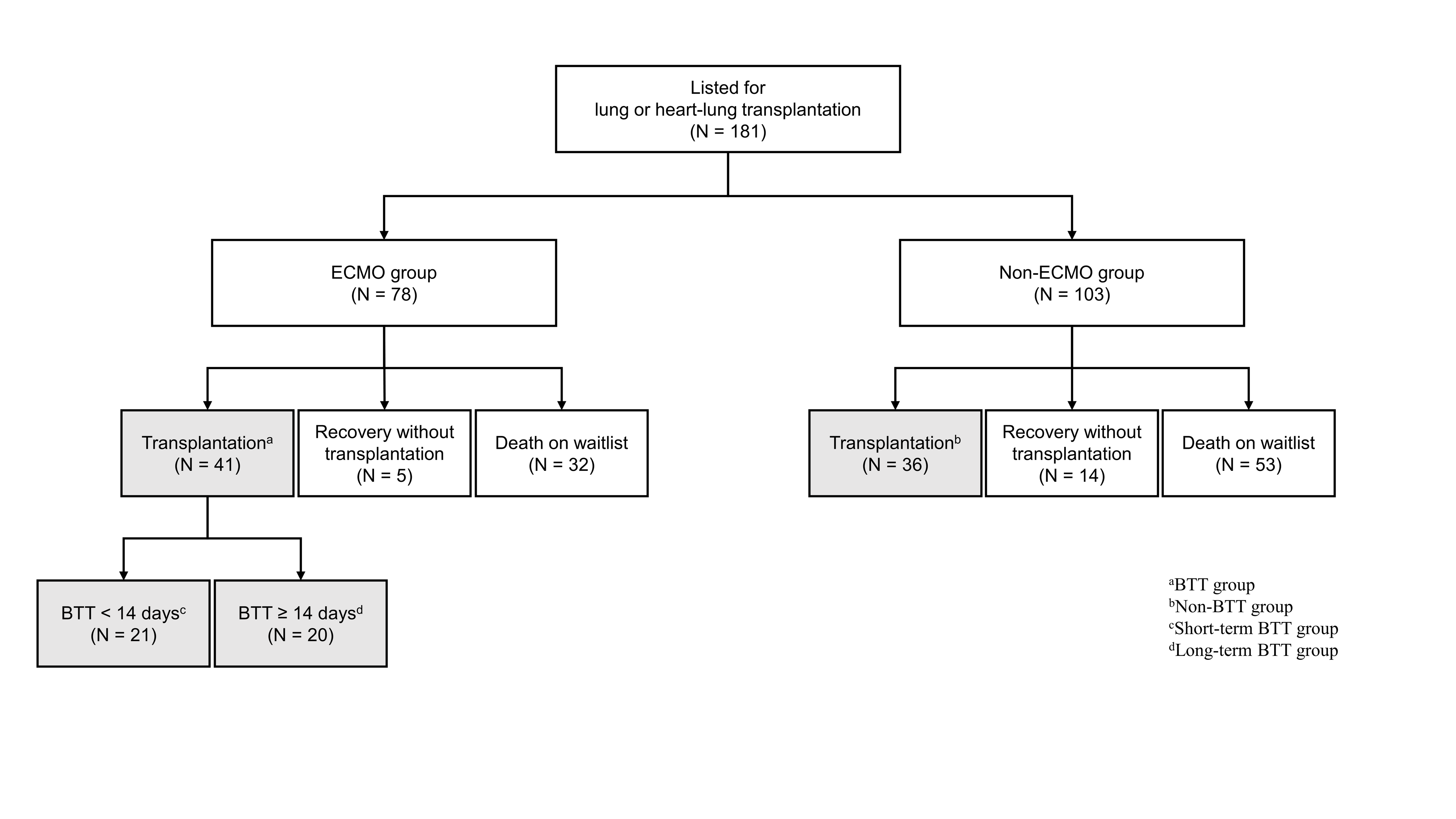

Supplement: S1 Fig — BTT, bridge to lung transplantation. aBTT group; bNon-BTT group; cShort-term BTT group; dLong-term BTT group. (TIF) [file pone.0253520.s001.tif]
