# Supplementary material for: Effects of the duration of bridge to lung transplantation with extracorporeal membrane oxygenation
Source: PLoS One. 2021 Jul 1;16(7):e0253520. doi: 10.1371/journal.pone.0253520 (PMC8248733; doi:10.1371/journal.pone.0253520)
Supplement: S1 Table — BTT, bridge to lung transplantation; CI, confidence interval; ECMO, extracorporeal membrane oxygenation; HR, hazard ratio; SAPS II, simplified acute physiologic score II. (DOCX) [file pone.0253520.s002.docx]

**Supplemental Digital Contents (SDC)**

S1 Table. Risk factors for 1-year post-transplant mortality in univariate analysis

(A) Univariate analysis

|  | HR | 95% CI | *P*-value |
| --- | --- | --- | --- |
| Age | 0.994 | 0.961–1.029 | 0.732 |
| Male | 0.800 | 0.310–2.065 | 0.645 |
| SAPS II | 1.016 | 0.993–1.039 | 0.171 |
| Immobilization (vs. mobilization) | 2.120 | 0.795–5.654 | 0.133 |
| BTT (vs. non-BTT) | 1.549 | 0.600–3.996 | 0.366 |
| BTT |  |  | 0.016 |
| Non-BTT | 1.000 |  |  |
| Short-term BTT (< 14 days) | 0.481 | 0.100–2.315 | 0.361 |
| Long-term BTT (≥ 14 days) | 3.070 | 1.141–8.260 | 0.026 |
| ECMO configuration at transplantation |  |  | 0.251 |
| Non-BTT | 1.000 |  |  |
| Veno-venous | 1.168 | 0.392–3.475 | 0.781 |
| Veno-arterial | 2.548 | 0.808–8.038 | 0.111 |

Abbreviations: BTT = bridge to lung transplantation; CI = confidence interval; ECMO = extracorporeal membrane oxygenation; HR = hazard ratio; SAPS II = simplified acute physiologic score II.
