# Supplementary material for: Effects of the duration of bridge to lung transplantation with extracorporeal membrane oxygenation
Source: PLoS One. 2021 Jul 1;16(7):e0253520. doi: 10.1371/journal.pone.0253520 (PMC8248733; doi:10.1371/journal.pone.0253520)
Supplement: S2 Table — BTT, bridge to lung transplantation; CI, confidence interval; ECMO, extracorporeal membrane oxygenation; HR, hazard ratio; SAPS II, simplified acute physiologic score II. aAdjusted for age, sex, and BTT. (DOCX) [file pone.0253520.s003.docx]

**Supplemental Digital Contents (SDC)**

S2. Table. Risk factors for 3-year post-transplant mortality

(A) Univariate analysis

|  | HR | 95% CI | *P*-value |
| --- | --- | --- | --- |
| Age | 1.001 | 0.970–1.032 | 0.972 |
| Male (vs. female) | 0.690 | 0.306–1.553 | 0.370 |
| SAPS II | 1.004 | 0.983–1.025 | 0.716 |
| Immobilization (vs. mobilization) | 1.582 | 0.653–3.833 | 0.310 |
| BTT (vs. non-BTT) | 0.968 | 0.435–2.155 | 0.936 |
| BTT |  |  | 0.085 |
| Non-BTT | 1.000 |  |  |
| Short-term BTT (< 14 days) | 0.413 | 0.117–1.464 | 0.171 |
| Long-term BTT (≥ 14 days) | 1.756 | 0.739–4.174 | 0.203 |
| ECMO configuration at transplantation |  |  | 0.575 |
| Non-BTT | 1.000 |  |  |
| Veno-venous | 0.782 | 0.308–1.988 | 0.606 |
| Veno-arterial | 1.449 | 0.510–4.119 | 0.487 |

Abbreviations: BTT = bridge to lung transplantation; CI = confidence interval; ECMO = extracorporeal membrane oxygenation; HR = hazard ratio; SAPS II = simplified acute physiologic score II.

(B) Multivariate analysis

|  | HR | 95% CI | *P*-value |
| --- | --- | --- | --- |
| Age | 1.017 | 0.979–1.056 | 0.380 |
| Male (vs. female) | 0.579 | 0.226–1.480 | 0.253 |
| BTT |  |  | 0.078 |
| Non-BTT | 1.000 |  |  |
| Short-term BTT (< 14 days) | 0.370 | 0.103–1.334 | 0.129 |
| Long-term BTT (≥ 14 days) | 1.670 | 0.700–3.986 | 0.248 |

Adjusted for age, gender (male), and BTT groups.

Abbreviations: BTT = bridge to lung transplantation; CI = confidence interval; HR = hazard ratio.
