# Supplementary material for: Effects of the duration of bridge to lung transplantation with extracorporeal membrane oxygenation
Source: PLoS One. 2021 Jul 1;16(7):e0253520. doi: 10.1371/journal.pone.0253520 (PMC8248733; doi:10.1371/journal.pone.0253520)
Supplement: S3 Table — BTT, bridge to lung transplantation; CI, confidence interval; ECMO, extracorporeal membrane oxygenation; HR, hazard ratio; SAPS II, simplified acute physiologic score II. aAdjusted for age, sex, and BTT. (DOCX) [file pone.0253520.s004.docx]

**Supplemental Digital Contents (SDC)**

S3 Table. Risk factors for 5-year post-transplant mortality

(A) Univariate analysis

|  | HR | 95% CI | *P*-value |
| --- | --- | --- | --- |
| Age | 1.007 | 0.76–1.038 | 0.657 |
| Male (vs. female) | 0.791 | 0.359–1.745 | 0.562 |
| SAPS II | 1.005 | 0.985–1.024 | 0.638 |
| Immobilization (vs. rehabilitation) | 1.623 | 0.701–3.759 | 0.258 |
| BTT (vs. non-BTT) | 1.124 | 0.520–2.433 | 0.766 |
| BTT |  |  | 0.080 |
| Non-BTT | 1.000 |  |  |
| Short-term BTT (< 14 days) | 0.551 | 0.178–1.710 | 0.303 |
| Long-term BTT (≥ 14 days) | 1.929 | 0.832–4.473 | 0.126 |
| ECMO configuration at transplantation |  |  | 0.766 |
| Non-BTT | 1.000 |  |  |
| Veno-venous | 1.003 | 0.422–2.381 | 0.995 |
| Veno-arterial | 1.440 | 0.506–4.096 | 0.494 |

Abbreviations: BTT = bridge to lung transplantation; CI = confidence interval; ECMO = extracorporeal membrane oxygenation; HR = hazard ratio; SAPS II = simplified acute physiologic score II.

(B) Multivariate analysis

|  | HR | 95% CI | *P*-value |
| --- | --- | --- | --- |
| Age | 1.021 | 0.983–1.059 | 0.285 |
| Male (vs. female) | 0.652 | 0.262–1.622 | 0.358 |
| BTT |  |  | 0.072 |
| Non-BTT | 1.000 |  |  |
| Short-term BTT (< 14 days) | 0.489 | 0.155–1.541 | 0.222 |
| Long-term BTT (≥ 14 days) | 1.841 | 0.790–4.292 | 0.158 |

Adjusted for age, gender (male), and BTT groups.

Abbreviations: BTT = bridge to lung transplantation; CI = confidence interval; HR = hazard ratio.
